# The Influence of Dehulling on the Nutritional Quality of Lupine Seeds (*Lupinus albus* L.) and the Effect of Their Use in the Feed of Laying Quails on the Live Performance and Quality of Eggs

**DOI:** 10.3390/ani11102898

**Published:** 2021-10-05

**Authors:** Dănuţ Ioan Struţi, Andrea Bunea, Ioan Mircea Pop, Tudor Andrei Păpuc, Daniel Pantea Mierliţă

**Affiliations:** 1 Department of Technological Science, Faculty of Animal Science and Biotechnologies, University of Agricultural Sciences and Veterinary Medicine Cluj-Napoca, 400372 Cluj-Napoca, Romania; ptudor2008@yahoo.com; 2 Department of Biochemistry, Faculty of Animal Science and Biotechnologies, University of Agricultural Sciences and Veterinary Medicine Cluj-Napoca, 400372 Cluj-Napoca, Romania; andrea.bunea@usamvcluj.ro; 3 Department of Animal Nutrition, Faculty of Animal Science, University of Agricultural Sciences and Veterinary Medicine of Iasi, 3 Mihail Sadoveanu Alley, 700490 Iasi, Romania; popmirceais@yahoo.com; 4 Department of Animal Science, Faculty of Environmental Protection, University of Oradea, 1 University Str., 410087 Oradea, Romania

**Keywords:** white lupine, dehulled seeds, laying quails, eggs, fatty acids, carotenoids

## Abstract

**Simple Summary:**

This research evaluated the influence of dehulling on the nutritional quality of *Lupinus albus* seeds and the effect of their utilization in the feed of laying quails on the live performance and quality of eggs. The dehulling of lupine seeds led to an improvement in nutritional value as a result of the increase in the protein and crude fat content and the reduction in crude fiber. The use of dehulled lupine seeds in the diet of laying quails improved the performance response and quality of eggs, compared with the use of whole seeds, with the results being similar to those obtained for the quails of the control group. The presence of lupine seeds in quail feed improved the fatty acids (FA) profile of yolk lipids by increasing the polyunsaturated FA proportion to the detriment of saturated FA. The quality of the yolk fats lipidic indices became more favorable to consumer health as a result of an increase in the FA proportion, with a corresponding hypocholesterolemic effect, and also as a result of a decrease in the atherogenic and thrombogenic index values.

**Abstract:**

*Lupinus albus* seeds from low-alkaloid varieties are a valuable alternative source of protein for reducing the dependence on soybean meal for the feeding of monogastric animals. Therefore, the aim of this research was to evaluate the dehulling influence of lupine seeds (*L. albus*, cv. Amiga) and the effect of their use in the laying quails feed on live performance and egg quality. A total of 200 laying quails (*Coturnix japonica*), with an age of 10 weeks, were randomly assigned to five experimental groups (five replicates/group). Each group was randomly assigned to one of five dietary treatments: the control group (C) diet was based on soybean meal, while the experimental groups received either 200 g/kg (WLS_20_) or 250 g/kg (WLS_25_) of whole lupine seeds in their diets, or 200 g/kg (DLS_20_) or 250 g/kg (DLS_25_) of dehulled lupine seeds in their diets. The results show that by dehulling the lupine seeds, the nutritional value of the seeds increased. The use of dehulled lupine seeds in the diet of laying quails did not affect the live performance (egg production, feed intake, feed conversion ratio), yolk cholesterol content, and physico-chemical quality indices of the eggs, compared with control. The presence of dehulled lupine seeds in the diet improved the nutritional quality of the yolk lipids because of the increase in the content of polyunsaturated fatty acids. Thus, the lipid quality indices of the yolk became more favorable to consumer health because of an increase in the h/H ratio (hypocholesterolemic/Hypercholesterolemic FA) and a decrease in the atherogenic index and thrombogenic index values. The higher content in carotenoids also contributed to the higher nutritional quality of the eggs obtained by lupine utilization. In conclusion, dehulling of lupine seeds had a positive influence on the nutritional quality of the seeds, the live performance of quails, and the quality of eggs.

## 1. Introduction

Intensive poultry feeding is dependent on conventional protein sources such as soybean meal, which has a high protein content (42–46%) with a balance of essential amino acids that corresponds to the nutritional requirements of birds. However, in the context of restriction of animal meal inclusion in poultry feed, the limited ecological possibilities of cultivating soybeans in many regions, and the tendency to limit the use of genetically modified soy in animal feed, it is necessary to evaluate unconventional sources of protein with high biological value that can be obtained locally and at lower cost than soy [1,2,3].

White lupine seeds from low-alkaloid varieties can be a suitable alternative to soybean meal, both nutritionally and economically, as well as in terms of food safety [4,5]. Soybean meal in poultry feeds are used in a 20% to 33% range, depending on the species and performance. Worldwide, there are four species of lupine that have been successfully cultivated: *Lupinus albus* and *Lupinus luteus* in Europe, *Lupinus angustifolius* in Australia, and *Lupinus mutabilis* in America [3,6]. Progress has been made by creating new varieties of sweet lupine, free of alkaloids (less than 0.02%) [7]. However, the nutrient content of lupine seeds and their quality are considerably influenced by species, variety, applied agrotechniques, and pedoclimatic conditions of plant growing [8,9]. Different from soybean, new varieties of sweet lupine (low-alkaloid) do not require heat treatment to remove thermolabile compounds because of their low level of protease inhibitors, tannins, saponins, and lectins. Among the lupine species, the utilization of *L. albus* seeds from low-alkaloid varieties are of great interest in poultry nutrition either because of their high content of crude protein (35–43%) and fat (8–12%) [6,10] or because of the quality of their essential amino acid and fats profile, especially their unsaturated fatty acids (70–75% of FAME) [11,12,13]. The high content of crude fiber of lupine (12–15%), raffinose family oligosaccharides (8.5%), and non-starch polysaccharides (NSPs, 29.5% of DM) such as arabinose, xylose, mannose, or galactose can decrease the nutritional value of feed, especially for poultry [14,15]. Soluble fiber binds large amounts of water during fermentation in the gastrointestinal tract (GIT) of birds, causing an increase in digestive viscosity, creating a gel-like consistency that reduces the absorption of feed nutrients [16]. Naturally, birds do not possess endogenous enzymes for NSP utilization [17].

Whole lupin has been shown to support proper performance, whether it is included in amounts up to 20% for laying hens, 25% for broiler chickens, and 18% for turkeys [18,19,20,21,22,23,24]. Conventional thermal processing methods such as cooking, autoclaving, extrusion, and germination have been widely utilized to inactivate the antinutritional factors in the grains used in poultry feeds. Despite being effective in most other ingredients, such methods have proven to be ineffective in lupin seeds [25,26,27,28,29,30]. Dehulling consists of the removal of hulls from whole seeds by mechanical processes. By dehulling lupins, approximately 60–70% of the crude fiber content is reduced, including non-starch polysaccharides [31,32,33], which may potentially increase seed nutritional values. Few studies have addressed the effect of the dehulling process on the nutritional quality of *L. albus* seeds for poultry. By dehulling lupins, broiler growth performance (body weight, feed conversion ratio) may be potentially increased by improving the nutrients’ digestibility (in terms of starch and amino acids coefficients) [34,35,36,37]. Little attention has been given to understanding the effects of dehulled lupine seeds on the performance and egg quality of laying hens and quail. In this regard, some research shows that dehulled *L. albus* seeds used in the diet of laying hens at quantities of 180 g/kg do not influence the laying rate, feed conversion ratio, and egg weight [38]. Similar results were subsequently obtained by Lee et al. [20] after including dehulled blue lupine seeds in the amount of 150 g/kg in the feed of laying hens.

To the best of our knowledge, there is just one study in which dehulled white lupine seeds were investigated, which was in Japanese quail feeds, and said study focused on the growth performance (body weight, feed intake, and feed conversion ratio) of birds before sexual maturity [38]. Worldwide, the quail egg production industry has been increasing due to the high productivity of birds and the nutritional quality of their eggs [39,40,41,42,43]. In the period 2008–2017, there was a 25% increase in the worldwide consumption of quail eggs, with an average consumption of about 30 eggs/capita/year. However, quail eggs represent only 3.2% (approx. 300 g/capita/year) of total worldwide egg consumption [44]. Given such background, we believe it to be opportune to investigate Japanese quail response to lupin during the egg laying cycle. The current research was conducted to determine the influence of dehulling on the nutritional quality of lupine seeds (*L. albus*, cv. Amiga) and the effect of their inclusion in feeds for Japanese quail on performance and egg quality.

## 2. Materials and Methods

### 2.1. Lupine Seeds, Chemical Composition and Nutritional Quality Analysis

The *Lupinus albus* (cv. Amiga) seeds used in this research belong to the low-alkaloid variety (less than 0.02%). The vegetal biological material (*L. albus* seeds) was purchased from a local farmer who cultivated this variety in 2018 in climatic conditions specific to the Transylvanian area (47°17′03″ N; 23°40′34″ E), Romania. The dehulled lupine seeds were obtained by mechanical removal of the hulls by a dehuller machine used in the food industry for other types of seeds (YMTP-25 dehulling machine Lushan Win Tone Manufacture Co., Ltd, Lushan, Henan, China). Laboratory samples were collected from both whole lupine seeds (WLS) and dehulled seeds (DLS) to perform the analyses of raw chemical composition, fatty acid content, and amino acid profile. The samples were ground with a Retsch laboratory mill type Pm100 (RETSCH GmbH manufacturer, Haan, Germany) and passed through a sieve with 0.5 mm holes.

The raw chemical composition analysis was performed according to the procedures established by AOAC International [45], the following being determined: dry matter (DM) (Method 934.01); crude ash (CA) by calcination (Met. 942.05); crude protein (CP) by the Kjeldahl method (Met. 954.01); crude fat as ether extract (EE) by the Soxhlet method (Met. 920.39); crude fiber (CF) after an acid hydrolysis followed by basic hydrolysis (Met. 978.10); organic matter (OM = 100 − CA) and nitrogen free extract (by difference: NFE = 100% − CP% + CA% + EE% + CF%). Data on the raw chemical composition of whole and dehulled lupine seeds are reported on a dry matter basis (% of DM) and are presented as an average (*n* = 5). Nitrogen-corrected metabolizable energy (AME_N_) of lupine seeds was calculated according to the formula proposed by Sibbald [46]: AME_N_ = 3951 + 54.4 MG − 88.7 CB − 40.8 Ce; where MG = crude fat, CB = crude fiber, Ce = crude ash.

The determination of the protein content in amino acids was performed by the instrumental analysis of high performance liquid chromatography (HPLC), in accordance with the standard of SR EN ISO 13903: 2005 (regarding the determination of free and total amino acids from feeding stuffs) and the EC 152/2009 regulation (establish the sampling and analysis methods for official feed control). The analyses were performed in the laboratories of the SCIENT Research and Instrumental Analysis Institute—Cromatec Plus from Snagov (Bucharest). Identification and quantification of the amino acids was performed using the Perkin Elmer LC 300 UHPLC system (PerkinElmer, Waltham, MA, USA). The work method steps involved preparation of lupine seeds samples (*n* = 5 WLS and *n* = 5 DLS), followed by hydrolysis with hydrochloric acid (c = 6 mol HCl/l; 2 mL/sample) and preparation of the amino acid standard stock solutions, injection of the standard solutions and drawing the calibration curves, followed by injection of lupine samples (vol. 10 µL), chromatogram analysis, and results calculation. Methionine was determined as methionine sulfone. Tryptophan was determined as total tryptophan by basic hydrolysis with saturated barium hydroxide. The identification of all amino acids (AAs) was performed by comparing the retention times of each AA in the standard used with those resulting from lupine samples (Santa Cruz Biotechnology standard: L-Lysine (CAS 56-87-1), L-Methionine (CAS 63-68-3), L-Tryptophan (CAS 73-22-3), Histidine (CAS 71-00-1), Valine (CAS 72-18-4), Phenylalanine (CAS 63-91-2), L-Isoleucine (CAS 73-32-5), L-Leucine (CAS 61-90-5), Arginine (CAS 74-79-3), Serine (CAS 56-45-1), Aspartic acid (CAS 56-84-8), Glutamine (CAS 56-85-9), Proline (CAS 147-85-3), Alanine (CAS 56-41-7).

Fatty acids from whole and dehulled lupine seeds (*n* = 5) were identified as methyl esters of fatty acids (FAME) (g/100 g of total FAME identified) using the gas chromatography technique with mass spectrometry detection, according to SR EN ISO/TS 17764-2: 2008 (specifies the application of gas chromatography determination of the quantitative content of fatty acids of animal and vegetable fats and oils) and ISO 5508: 2002 (general guidance for the application of gas chromatography to determine the qualitative and quantitative composition of a fatty acid methyl esters mixture). The principle of the method consisted of the chromatographic separation of the fatty acid mixture from lupine oil initially esterified with sodium metanoate 0.5 mol/L, on a sand bath at 210 °C and a reflux rate of 1 drop/second, for up to 30 min, followed by the introduction of 5 mL of trifluoride solution of boron on a capillary column with low polar stationary phase, followed by fragmentation of their molecules by electronic impact in the ionization source. The method first involved the fats saponification, followed by esterification under a catalyst of boron trifluoride 15% vol. The equipment used included a Perkin Elmer Chromatographic system with a mass spectrometer detector (GC-MS) (gas chromatograph Clarus 680 + spectrometer table Clarus SQ8T quadrupole). The chromatographic column was Elite-Wax, 30 m length, 0.25 mm internal diameter, and 1.0 μm film thickness. The injection port temperature was 220 °C, the injected sample volume was 1.0 μL, and the carrier gas was helium at a flow rate of 1.5 mL/min, splitting ratio 40:1. The identification of the fatty acid peaks obtained from the samples was performed by comparing the relative retention time of FAME with that of the certified standard (Mix FAME Supelco 37).

### 2.2. Quails, Experimental Design and Diets

A total of two hundred 8-week-old laying Japanese quails (*Coturnix japonica*) were randomly assigned to five experimental groups (treatments), with five replicates of 8 quails (40 quails/group). The quails were kept in standard battery cages, according to the regulations for poultry maintenance (Directive 98/58/EC), with an equal surface area of 337.5 cm^2^/quail. Each battery cage was individually equipped with nipple drinkers and feed trough, trays for manure collection, and a gutter for egg collection; therefore, the maintenance of technological conditions was identical for each bird, replicate, and experimental group.

There were five experimental diets randomly assigned to each experimental group. The composition and nutritional characteristics of the tested diets are presented in Table 1.

All the tested diets were formulated to ensure the standard nutritional requirements of laying quails according to NRC [47]. Experimental treatments consisted of control feed formulated with soybean meal as the main source of protein and four diets in which whole (WLS) or dehulled (DLS) lupine seeds were included at 200 g/kg or 250 g/kg. The quails had ad libitum access to the water source and feed.

**Table 1:** Composition and nutritional characteristics of the diets used in the laying quails feeding.

Specification	^1^ C	Experimental Diets			
		^2^ WLS_20_	^3^ WLS_25_	^4^ DLS_20_	^5^ DLS_25_
Composition of feed (%)					
Maize (8.0% Cp)	46.03	41.85	41.10	49.03	49.75
Triticale (11.4% Cp)	10.00	10.00	10.00	10.00	10.00
Soybean meal (46% Cp)	33.00	16.50	12.30	12.00	6.80
Lupine whole seeds	-	20.00	25.00	-	-
Lupine dehulled seeds	-	-	-	20.00	25.00
Sunflower oil	3.20	3.85	3.80	1.10	0.50
DL-Methionine	0.02	0.05	0.05	0.05	0.05
L-lysine HCl	-	-	-	0.07	0.15
Limestone	5.25	5.25	5.25	5.25	5.25
Vitamin–Mineral premix ^6^	2.50	2.50	2.50	2.50	2.50
TOTAL	100.00	100.00	100.00	100.00	100.00
Nutritional characteristics (calculated values)					
Metabolizable energy (kcal/kg)	2901	2906	2904	2903	2903
Crude protein (%)	20.02	20.04	20.03	20.02	20.04
Ether extract (%)	5.83	7.90	8.23	5.55	5.42
Crude fiber (%)	2.88	4.55	4.98	2.66	2.61
Lysine (%)	1.02	1.03	1.01	1.00	1.00
Methionine (%)	0.45	0.45	0.45	0.45	0.45
Methionine + cysteine (%)	0.80	0.81	0.81	0.83	0.83
Calcium (%)	2.50	2.50	2.50	2.50	2.50
Available phosphorus (%)	0.43	0.44	0.44	0.43	0.43

^1^ C, control diet, without lupine; ^2^ WLS_20_, experimental diet with 200 g/kg whole lupine seeds; ^3^ WLS_25_, experimental diet with 250 g/kg whole lupine seeds; ^4^ DLS_20_, experimental diet with 200 g/kg dehulled lupine seeds; ^5^ DLS_25_, experimental diet with 250 g/kg dehulled lupine seeds. Diets formulated according to NRC [48]. ^6^ Vitamin–Mineral premix: vitamin A 480.000 IU/kg, vitamin B1 60 mg/kg, vitamin B2 200 mg/kg, vitamin B4 16.800 mg/kg, vitamin B5 441 mg/kg, vitamin B6 200 mg/kg, vitamin B9 60 mg/kg, vitamin B12 0.6 mg/kg, vitamin D3 95.000 IU/kg, vitamin E 1.200 IU/kg, vitamin H 4 mg/kg, vitamin K3 100 mg/kg, vitamin PP 2.400 mg/kg, copper 600 mg/kg, iron 2.160 mg/kg, iodine 48 mg/kg, manganese 3.720 mg/kg, selenium 6 mg/kg, zinc 2.844 mg/kg, calcium 145 g/kg, phosphorus 123 g/kg, chlor 7.1%, sodium 5.5%; DL-Methionine 54.72 g/kg, BHT, propil galat (E310), etoxiquin.

Throughout the experimental period, average temperature, humidity (monitored with a digital thermo-hygrometer), and air ventilation speed recorded in the experimental facility were 22.0 °C (±0.4), 70.0% (±0.8), and 0.2 m/s, respectively. The daily lighting regime ensured was 18 h of light and 6 h of darkness. Before the start of the experiment, a pre-experimental period of 2 weeks was carried out, in which the quails were weighed and relocated on replicas and groups. Thus, at the beginning of the experiment, there was equality and uniformity between the experimental groups. The experimental period lasted for 8 weeks, from the age of the quails of 10 weeks to 18 weeks of age.

### 2.3. Performance Responses

At the beginning and the end of the experiment, the body weight (g) of the quails from each treatment group was measured (*n* = 8 birds/replicate and 40 birds/group). Egg production was recorded daily to calculate rate of laying (%), and weekly the eggs laid on two consecutive days were weighed individually and egg mass production was calculated. Feed intake (FI) was monitored weekly according to the replica and experimental group (g of feed/quail). Feed conversion ratio (FCR) was measured weekly per egg mass (kg feed/kg egg mass) and per dozen of eggs (kg feed/dozen). There was no mortality during the assays period. The health and behavior of quails were monitored daily.

### 2.4. Egg Quality Measurements

The eggs were collected and weighed weekly with an analytical balance. Egg weight was obtained from the eggs laid in the last two days of each 7 d interval.

The morphological structure of the egg components was performed bi-weekly at 2, 4, 6, and 8 w by weighing the yolk, eggshell, and the albumen (determined as the difference between: whole egg − (yolk weight + eggshell weight)) from a number of five eggs/replicate (25 eggs/group). The eggs were collected in the morning and selected based on the average egg weight of the experimental unit. The morphological components were analyzed and expressed as percentage of the whole egg: % albumen, % yolk, % eggshell. Values are presented as mean of data obtained from each evaluation from the entire experiment.

The physical egg quality indices such as albumen index (AI % = equatorial diameter ÷ height × 100), yolk index (YI % = equatorial diameter ÷ height × 100), and albumen Haugh unit (HU = 100 log (height − 1.7) × egg weight^0.37^ + 7.57) [48] were assessed on five fresh eggs (*n* = 5/replicate and 25/group) at the beginning and at the end of the experiment. The eggshell thickness was assessed with a Stainless electronic caliper (accuracy 0.01 mm), after the shells were dried in the oven and the internal membrane was removed. Eggshell thickness is expressed as a mean of three evaluations performed in three different regions: rounded peak, sharp peak, and median area. The yolk color was assessed using the color as determined by the La Roché scale, 1–15 points.

### 2.5. Chemical Analysis and Nutritional Quality of the Eggs

The raw chemical composition of eggs was performed on the eggs collected from each replicate and group in the last two days of the experimental period. The chemical content was performed according to the AOAC International procedures [45] described above (the crude protein and ash of the albumen and the crude fat, crude protein, and ash of the yolk). The fatty acids profile and cholesterol content (*n* = 5) was determined from the egg yolk lipids. The analyses of raw chemical content, cholesterol, carotenoids, and fatty acids profile were performed only from the eggs of the C, WLS_20_ and DLS_20_ groups, due to the higher performance responses obtained, which justified the nutritional quality analyses.

The cholesterol content of egg yolk was determined in accordance with AOAC International [49] Official Method no. 994.10 and the Method no. 976.26 and according to Horwitz [50]. The principle of the method consists of the saponification of samples, followed by petroleum ether extraction; afterwards, the concentration is resumed in chloroform. Standard solution of cholesterol in chloroform 10 mg/mL was used [51]. A Perkin Elmer-Clarus 500 gas chromatograph was used with flame ionization detector and HP-5 capillary separation column, 30 m × 0.320 mm diameter, film thickness 0.10 μm, H_2_ carrier gas, and the flue gas—air.

The fatty acid content of the yolk fats of quail eggs from the C, WLS_20_, and DLS_20_ groups was performed by extraction with chloroform–methanol, according to the method described by Folch et al. [52], followed by gas chromatography analysis. The concentration of fatty acids is expressed in grams of fatty acids methyl esters (FAME)/100 g total FAME identified (*n* = 5). The method used was in accordance with: SR EN ISO 5508/2002, SR EN ISO 5509/2002, and SR EN ISO 15,304/AC 2005. The principle of the fatty acids extraction method consisted of the transformation of fats into methyl esters of fatty acids using the acid-catalyzed transesterification procedure described by Christie [53]. Further work steps involved the separation of FAME in the chromatographic column, their identification by comparison with the standard chromatograms of the certified standards used (FAME mix, SUPELCO, no. 47885-U), and the quantitative determination of fatty acids. The equipment used was a Clarus 500 gas chromatograph (PerkinElmer Life and Analytical Sciences 710 Bridgeport Avenue Shelton, CT, USA) equipped with a capillary column injection system, equipped with a flame ionization detector (FID) and a capillary separation column type BPX70 with stationary phase with medium or high polarity. The column length was 60 m × 0.25 mm and 0.25 µm film thickness interior diameter. Special gases used: H_2_—carrier gas and air—flue gas.

In order to highlight the influence of the dietary treatments on the nutritional qualities of yolk fats, it was considered optimal to calculate some sanogenic lipid indices (*n* = 5), as follows:

The ratio between the omega-6 and omega-3 unsaturated fatty acids series: *n*-6/*n*-3.

Polyunsaturation index (PI) of yolk fats according to the equation proposed by Timmons [54]:

PI = C18: 2 *n*-6 + (C18: 3 *n*-3 × 2) (1)

Relevant Indices for human health, such as the atherogenic index (AI) and throm-bogenic index (TI) of lipids, according to Ulbricht et al. [55]:

AI = (C12:0 + C16:0 + 4 × C14:0) ÷ [ΣMUFA + Σ (*n*-6) + Σ (*n*-3)] (2)

TI = (C14:0 + C16:0 + C18:0) ÷ [0.5 × ΣMUFA + 0.5 × Σ (*n*-6) +3 × Σ (*n*-3) + Σ (*n*-3) ÷ Σ (*n*-6)] (3)

The ratio between the fatty acids with hypocholesterolemic (h) and hypercholesterolemic (H) effect was calculated using the equation of Fernandez et al. [56]:

h/H (hypocholesterolemic/Hypercholesterolemic) = (C18:1 + PUFA) ÷ (C12:0 + C14:0 + C16:0)(4)

The health promotion index (HPI) was calculated according to the equation proposed by Chen et al. [57]:

HPI = (*n*-3 PUFA + *n*-6 PUFA + MUFA) ÷ [C12:0 + (4 × C14:0) + C16:0)] (5)

### 2.6. Carotenoids Content of Egg Yolk

The carotenoids were extracted from 5 g egg yolk according to the procedure described by Schlatterer et al. [58]. The yolks were mixed and extracted with a mixture of methanol/ethyl acetate/petroleum ether (1:1:1) for three times. The residue obtained was placed in a separatory funnel with water, saline solution, and diethyl ether. The obtained ether phase was evaporated to dryness and diluted with a solution of TBME/methanol. It was filtered and subjected to high-performance liquid chromatography analysis (HPLC-PDA). HPLC-PDA quantification of carotenoids was performed by a Shimadzu LC20 AT HPLC with a SPDM20A diode array detector. The HPLC equipment consisted of a C30 column (24 cm × 4.6 mm, size: 5 µm) and a gradient system consisting of two solvents: A (methanol/tert-butyl methyl ether/water) and B (tert-butyl methyl ether/methanol/water). The flow-rate was adjusted to 1.0 mL min^−1^. A 20 µL sample or standard was injected into the HPLC system. The DAD detector was set at 450 nm. Lutein, zeaxanthin, and β-cryptoxanthin standards were provided by LGC Standards (UK). The retention time and absorption spectrum were recorded in the range of 300–550 nm.

### 2.7. Statistical Analysis

The statistical analysis was performed using GraphPad Prism software version 8.3.0 (GraphPad Software Inc.; San Diego, CA, USA). The statistical effect of diet on the egg chemical composition, cholesterol, and carotenoids content, as well as the fatty acids profile and sanogenic lipid indices of the egg fats, were tested by one-way analysis of variance (ANOVA) at a significance level of 5%. The ANOVA single-way test was also used to analyze the effect of dehulling on the chemical composition of white lupine seeds. For variables with significant variation (*p* < 0.05), the Tukey HSD post hoc test was used to establish the differences due to the applied treatments. Differences were considered significant when *p* < 0.05 and highly significant when *p* < 0.001. The ANOVA test was performed for the performance response (weight of quails, feed intake, laying rate, feed conversion ratio) and physical parameters of the eggs, considering the main effect of the diet and week, as well as the interaction between these. The Tukey multiple-range test was used to compare the differences between the mean values of applied treatments. Differences were considered significant when *p* < 0.05. All data are expressed as mean ± standard deviation.

## 3. Results

### 3.1. The Effect of Dehulling on the Chemical Composition of White Lupine Seeds

The raw chemical composition of whole and dehulled *Lupinus albus* seeds (cv. Amiga) from low-alkaloid varieties is presented in Table 2.

Through dehulling, a higher level in crude protein (*p* < 0.01) and ether extract (*p* < 0.05) was obtained, simultaneously with the reduction in the crude fiber content (*p* < 0.01) (Table 2). In addition, dehulling led to an increase in their energy value (AME_N_) (*p* < 0.01).

Dehulling of white lupine seeds did not influence (*p* > 0.05) the sum of saturated fatty acids (ΣSFA) and unsaturated acids (ΣUFA) proportion in the fat structure of *L. albus* seeds (Table 3).

The fatty acid profile highlights the high presence of unsaturated fatty acids (on average 83% of FAME). The dehulling increased the proportion of monounsaturated fatty acids (MUFA) to the detriment of polyunsaturated fatty acids (PUFA) (*p* < 0.001) from the fats structure. Among the MUFA, the most abundant was oleic acid (C18:1 *n*-9), and, from the polyunsaturated ones, the α-linolenic acid (C18:3 *n*-3) had the higher content (Table 3).

The effect of dehulling the low-alkaloid white lupine seed on the amino acid content of proteins is presented in Table 4. The results show that white lupine seeds (cv. Amiga) had a high content of amino acids such as lysine, arginine, leucine, histidine, glutamine, proline, serine + aspartic acid, but were deficient in methionine and tryptophan (Table 4).

Dehulling of lupine seeds significantly increased (*p* ˂ 0.001) the concentration of amino acids in the proteins structure (Table 4). Some of the essential amino acids such as lysine, methionine, tryptophan and leucine had higher values (*p* ˂ 0.001) in dehulled seeds. Among the non-essential amino acids, there were high levels of glutamine and arginine.

### 3.2. Influence of Utilizing Dehulled White Lupine Seeds in the Diets of Laying Japanese quails

#### 3.2.1. Performance Response

The results regarding the effect of dehulling white lupine seeds used in quail feed on the performance response are presented in Table 5.

The partial replacement of soybean meal in the laying quail diets with white lupine seeds led to a decrease in body weight during the experimental period (*p* < 0.05), with the exception of quails in the treatment with 200 g dehulled lupine seeds/kg feed (DLS_20_). In this case, the body weight of birds recorded at the end of the experiment was similar (*p* > 0.05) to that obtained in the control group (C).

The use of 250 g whole lupine seeds/kg feed determined a decrease in the laying rate (*p* < 0.05) compared with the control (C), but by dehulling lupine seeds, the laying rate increased, being similar to that of quails from control. Including the lupine seeds in the quail diet in the amount of 200 g/kg feed, both of whole (WLS_20_) and dehulled (DLS_20_) seeds did not affect the laying rate, although by dehulling there was a tendency to increase the egg production, without being statistically significant (Table 5).

Daily feed intake (mean g of feed/quail) was not influenced by the dietary treatments (*p* > 0.05). Therefore, the quails with the lowest egg production (LWS_25_) had the highest value of feed conversion ratio (*p* < 0.05) (Table 5). A high content of whole lupine in diet (250 g/kg) led to the depreciation of both feed conversion as kg feed/kg egg mass and as kg feed/dozen. Dehulling of lupine seeds led to an improvement in FCR that was similar to that obtained in the control group (Table 5).

**Figure 1:**
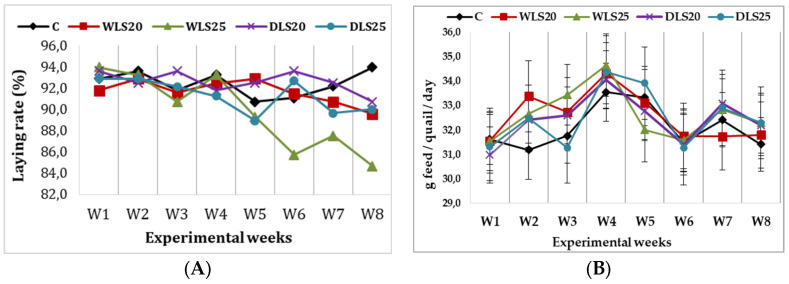
The influence of dehulling white lupine seeds on the laying rate evolution (%) (**A**) and daily feed intake of quails (g/quail/day) (**B**). W1–W8, experimental weeks; C, control group, without lupine; WLS_20_, experimental diet with 200 g/kg whole lupine seeds; WLS_25_, experimental diet with 250 g/kg whole lupine seeds; DLS_20_, experimental diet with 200 g/kg dehulled lupine seeds; DLS_25_, experimental diet with 250 g/kg dehulled lupine seeds.

The dynamics of the laying rate evolution and the average daily feed consumption during the experimental weeks (1–8 weeks) are presented in Figure 1.

There were no mortalities or any visible changes in the health of the quails during the experimental period.

#### 3.2.2. Egg Quality Traits

The partial replacement of soybean meal with whole lupine seeds introduced in the amount of 200 or 250 g/kg feed in the laying quail diets determined a decrease in egg weight (*p* < 0.01) compared with the control group. In addition, by dehulling lupine seeds, the egg weight increased, being similar to that recorded in the control (*p* > 0.05) (Table 6).

Seed dehulling determined an increase in egg weight (*p* < 0.05) compared with the use of whole seeds only when lupine was introduced in the amount of 200 g/kg feed (DLS_20_ vs. WLS_20_) (Table 6).

Dietary treatments did not influence (*p* > 0.05) the albumen and yolk percentages of the whole egg structure (Table 6). The use of whole seeds led to a decrease in the eggshell weight from the egg structure (*p* < 0.05), but seed dehulling had a positive effect on this quality parameter of the eggs (Table 6). The result of dehulling was also reflected in eggshell thickness (mm), which decreased (*p* < 0.05) simultaneously with the increase in the proportion of the lupine seed inclusion in the diet (Table 6), the effect being more accentuated when whole seeds were used. Dehulling of lupine increased shell thickness, but it was lower compared with the control group (*p* < 0.05).

Dehulled white lupine used in quail feed did not influence the determined physical quality indices of the eggs, such as albumen index (*p* > 0.05), yolk index (*p* > 0.05) and Haugh unit of the albumen (*p* > 0.05) (data unpresented).

#### 3.2.3. Egg Chemical and Nutritional Composition

Dehulling of lupine seeds did not influence (*p* > 0.05) the albumen content in crude protein and crude ash, and neither did it influence the yolk content in crude fat, protein and ash, compared with control and WLS_20_. However, there was a higher level of crude fat in egg yolk from the quails in DLS_20_ compared with the control (C) and WLS_20_ (63.51% vs. 59.48–58.98% of DM), but the differences were not statistically supported (data unpresented).The partial replacement of soybean meal with lupine seeds and the dehulling of lupine did not influence the cholesterol content (*p* > 0.05) of quail eggs, even if it had a tendency to decrease, especially when dehulled seeds were used (data unpresented).

The quality of fats was represented by the fatty acids profile, their concentration, and the ratio between them. In all cases the yolk fats of the analyzed quail eggs, the fatty acids with the best representation were oleic acid (32.26–34.66% of FAME), palmitic acid (21.38–25.84% of FAME), and linoleic acid (12.49–16.31% of FAME) (Table 7).

The inclusion of white lupine seeds in the laying quail diets produced important changes in the fatty acid profile of yolk fats (Table 7). Compared with the group without lupine (C), the egg yolk from the groups with lupine-fed quails (WLS_20_ and DLS_20_) contained less (*p* < 0.05) saturated fatty acids, but was richer (*p* < 0.05) in monounsaturated fatty acids (especially in C18:1 *n*-9 and C16:1) and in polyunsaturated fatty acids (especially C18:3 *n*-6 and C20:4 *n*-6) (Table 7).

Dehulling of lupine seeds and their use in quail feed led to a decrease in the SFA proportion in the fats structure of egg yolks (*p* < 0.05) and an increase in UFA content, compared with the use of whole seeds (Table 7). In addition, the concentrations of palmitoleic, heptadecanoic, α-linolenic, docosapentaenoic, and docosahexaenoic fatty acids from the egg yolk increased (*p* < 0.05), but the concentrations of nervonic and docosatetraenoic fatty acids decreased (*p* < 0.05). Dehulling of lupine seeds and their use in the quails diet (DLS_20_) determined an increase (*p* < 0.05) in hypocholesterolemic fatty acids (hFA) proportion in the egg yolk, compared with the group of quails that received whole seeds (DLS_20_) (59.72% vs. 52.74% of FAME) (Table 7).

The nutritional quality of the fats from egg yolk, assessed on the basis of sanogenic lipid indices, showed that the use of white lupine seeds (whole and dehulled) in quail diets led to obtaining sanogenic indices more favorable for consumer health (Table 8). In this sense, the results show an increase in the value of the health promotion index (HPI) when lupine seeds were included in the quail diets, the differences being statistically supported after dehulling lupine seeds (C < WLS_20_ < DLS_20_).

Compared with the control group (C), the use of lupine seeds determined an increase (*p* < 0.05) in the h/H index value, which shows the ratio between fatty acids with hypocholesterolemic effect (h) and hypercholesterolemic effect (H). The differences were statistically significant after using dehulled seeds (DLS_20_) (2.06 vs. 2.80) (Table 8).

The use of lupine seeds in the quails diet improved (*p* < 0.05) the value of the indices that highlight the predisposition for the incidence of cardiovascular diseases—namely, the atherogenic (AI) and thrombogenic (TI) indices of the fats from yolk—compared with the group without lupine (Table 8).

#### 3.2.4. Carotenoids Content of Egg Yolk

The influence of white lupine seeds usage in the quail diets on the yolk carotenoids content is presented in Table 9 The use of whole and dehulled lupine seeds in the quail diets in the amount of 200 g/kg feed led to an increase in the carotenoids content (*p* < 0.05) of quail egg yolk (13.91 vs. 22.80 and 18.63 µg/g fresh weight) (Table 9).

Dehulling of lupine seeds did not significantly influence (*p* > 0.05) the carotenoids level of egg yolk, although a decreasing tendency in the content could be noticed, without being statistically significant. In particular, lutein and zeaxanthin were found in higher concentrations (*p* < 0.05) in eggs of quails fed diets containing lupine seeds (whole and dehulled). Two other carotenoids detected in yolk—canthaxanthin and β-cryptoxanine—were found in higher concentrations (*p* < 0.05) in WLS_20_ than in the Control group (Table 9).

## 4. Discussion

Soybean meals are the main source of protein for poultry feed because of their high protein content, with a balanced amino acid profile. However, the worldwide increase in demand for soybean meal as a result of livestock increase has led to rising prices and to fluctuations in market supply [1,2,4]. Thus, it has become necessary to evaluate other plant sources of protein as potential substitutes for soybean meal in poultry feed. White lupine seeds of low alkaloid varieties are suitable for soybean meal substitution in poultry feed, because of their high protein level, balanced amino acid profile, and high content of unsaturated fatty acids [11,13]. White lupine is promoted as an alternative source of protein for poultry feed and, because of its low price compared with soybeans, it is available on the market. In addition, most lupine varieties are not genetically modified [23]. For these reasons, lupine cultivation is expanding in many parts of Europe. In Romania, the cultivation and use of lupine in poultry feed is not as common as in neighboring countries. In the present study, we evaluated the influence of dehulling on the nutritional quality of lupine seeds (*L. albus*, cv. Amiga) and the effect of inclusion in Japanese quails’ feed on the performance and quality of eggs. No studies have been performed on the effect of dehulling lupine seeds used in quail feed on the performance and quality of eggs.

### 4.1. Dehulled White Lupine Seeds

The effect of dehulling on the raw chemical composition of *L. albus* seeds (cv. Amiga) results in obtaining a higher level of crude protein (*p* ˂ 0.01) and ether extract (*p* ˂ 0.05), and a lower content of crude fiber (*p* ˂ 0.01). Such improvement in lupin nutritional quality may be attributed to hull removal that is rich in fiber and nitrogen-free extract. The effect of seed dehulling on lupine crude protein content obtained in this research agrees with the results reported by Písaříková et al. [32] in Butan cultivar (38.48% vs. 43.57% of DM), and Laudadio and Tufarelli [37] in Multitalia cultivar (35.50% vs. 42.93% of DM). For the ether extract content, the increase of 1.21% is comparable with the 2.3% increase obtained by Písaříková et al. [32], but it is much lower than the 6.5% increase reported by Saez et al. [33]. The decreases in the crude fiber level by 9.8% in dehulled seeds is similar to that obtained by Písaříková et al. [32] (11.5%) but also similar to that obtained by Saez et al. [33] at the cv. Hamburg (7.8%). The high crude fiber content of *L. albus* seeds exerts an antinutritional factor for monogastric animals, which cannot efficiently assimilate fiber because of the lack of specific endogenous enzymes [9]. Therefore, the nutritional value of lupine seeds for monogastrics can be considerably improved by dehulling [9,35]. The content of nitrogen-free extract (N-FE) did not change significantly by seeds dehulling, this result being in accordance with the research of Písaříková et al. [32] and Saez et al. [33]. The content in crude ash is relatively constant (*p* ˃ 0.05) after dehulling, with other research highlighting comparable results [32,33,37]. Dehulling of lupine seeds influences (*p* ˂ 0.01) the nitrogen-corrected metabolizable energy (AME_N_), an increase of 29.47% being obtained in dehulled seeds. In the literature, there are AME_N_ values reported for only whole white lupine seeds, which are in agreement with our results of 3326.9 kcal/kg DM for the cv. Energy and 2697.09 kcal/kg DM for cv. Lublanc [59].

The fats of *L. albus* seeds are characterized by a valuable fatty acid profile, given by the high concentrations of monounsaturated and polyunsaturated fatty acids. Used in farm animal feeds, white lupine seeds rich in unsaturated fatty acids contribute to obtaining animal by-products with valuable nutritional properties for human consumption [60,61]. According to some studies, the profile and fatty acids concentration from the lupine seed fats have different values depending on the species, genotype, cultivar, and pedoclimatic conditions [12,62]. Compared with our results, Musco et al. [10] obtained lower levels of palmitic (7.27% of FAME), oleic (46.60% of FAME), and α-linoleic acids (9.60% of FAME) but higher levels of linoleic (17.80% of FAME) and erucic acids (1.57% of FAME) in whole *L. albus* seeds. Seed dehulling influences the concentration of certain fatty acids such as oleic, linoleic, eicosenoic, and behenic acids. To our knowledge, there are few studies that have analyzed the effect of dehulling lupine seeds on the fatty acid profile of fats. For example, Suchý et al. [63] showed an increase (*p* ≤ 0.01) in the polyunsaturated and saturated fatty acids level (at *L. albus*, *L. angustifolius*, *L. luteus*) of 20.04–25.18% on average, a finding that was not observed in our research. More recently, Volek et al. [64] reported values of saturated and polyunsaturated fatty acids that were considerably lower than those obtained in this research for dehulled white lupine (cv. Zulika) but that were higher for monounsaturated fatty acids.

The concentration and amino acids profile of lupine seed proteins is variable depending on the factors that influence other nutrients [10,65]. In this research, the effect of dehulling resulted in increasing by 0.04–0.74% the concentration of main essential amino acids from lupine seed proteins, and increasing by 0.64–4.98% non-essential amino acids. Similar results were found by Mera-Zúñiga et al. [66], who obtained an increase in the level of all essential amino acids of 0.07–0.48% through dehulling blue lupine seeds. Similar results were presented by Laudadio and Tufarelli [37] for *L. albus*, and by Nalle et al. [35] for *L. angustifolius*.

### 4.2. Performance Responses of Quails

In the current research, quails that were fed dehulled lupin seeds of up to 200 g/kg feed exhibited similar performance response as the birds that were fed control feeds. The impairments noticed in performance response (final body weight, laying rate, average egg weight, and feed conversion ratio) of quails from DLS_25_ and especially WLS_25_ groups may have been due to the higher soluble NSP content of lupine diets. Soluble NSP exerts an anti-nutritional effect for poultry, primarily through increasing viscosity of the intestinal content and decreasing the digestive enzymes’ contact with the substrates of the intestinal tract, reducing nutrient digestion and absorption [67]. On the other hand, the high NSPs content of lupine-rich diets contributed to decreasing the amount of available energy from feed, as the negative relationship between NSPs content and efficiency of feed energy utilization in poultry is known [67]. Thus, we assume there was a lower amount of extra energy that needed to be stored in the body as fat in the case of groups fed lupine-rich diets, which may explain the differences in body weight. A lower body weight of laying hens that received 240 g/kg of whole white lupine seeds in the feed compared with those of the control was reported by Kubis et al. [21]. However, Rutkowski et al. [68] showed that an amount of up to 250 g/kg of whole yellow lupine seeds in the diets of laying hens did not produce significant changes in the final body weight of birds, compared with the group without lupine. Other studies presented body weight values for laying Japanese quails ranging between 211.5 and 371.1 g [69,70,71,72], an interval where the quail weights from the present research are situated.

In our research, the performance response of quails from WLS_20_ and DLS_25_ groups were similar; therefore, it was possible to increase the lupine inclusion in the laying quails diet from 200 g to 250 g/kg feed by dehulling, successfully reducing the amount of soybean meal used in feed from 16.5% (% of weight—WLS_20_) to 6.8% (DLS_25_); thus, a decrease of 41.2% of the soybean meal was obtained. Compared with the control group, the seeds’ dehulling and their utilization in the laying quail diets allowed a reduction in soybean meal proportion of 63.6% (in the case of DLS_20_) and of 79.4% (in the case of DLS_25_). Therefore, the level of soybean meal in feed composition decreased from 33.0% (C) to 12.0% and 6.8%, respectively (in the case of DLS_20_ and DLS_25_), without affecting the laying rate, the egg weight, and the feed conversion ratio.

Laudadio and Tufarelli [37] used dehulled *L. albus* seed in the amount of 180 g/kg in laying hen diets and reported that the performance (body weight at the end of the experiment, feed conversion kg of feed/kg egg, laying rate, and egg weight) was similar to that obtained for the hens from the group without lupine. The results obtained by the quails that received whole lupine seeds in a proportion of 20% in the feed are in agreement with the findings of Park et al. [73], who showed that blue lupine seeds utilization up to 22% in the diet of laying hens provided an even higher egg production than hens that received only soybean meal. Other research, in accordance with our data, also showed that positive performance was obtained for laying hens in terms of body weight, feed intake, egg production (%), and feed conversion ratio (kg feed/kg egg mass and kg feed/dozen egg) when whole blue lupine [19,20,74] or yellow lupine [60] seeds were included in the feed structure up to 20%. On the other hand, when whole white lupine [21] and yellow lupine seeds [68] were used in proportions higher than 24% in laying hen diets, the laying rate decreased, and the feed conversion ratio increased significantly because the substitution of soybean meal with lupine became progressively higher-observations that were encountered in the present research. On the contrary, Krawczyk al. [60] showed that hen weight, laying rate and feed conversion ratio (kg feed/kg eggs) were not significantly affected even if whole yellow lupine seeds were included in the amount of 300 g/kg in feed.

The decrease in performance response registered for quails from the WLS_25_ group was assumed to occur due to the high level of cell wall compounds from the hulls, combined with the presence of non-starch polysaccharides in the kernel, being known for their negative influence on the digestion and absorption of nutrients from poultry feed [14,22]. Results similar to ours (egg intensity, feed consumption, FCR, egg weight) have been previously reported in the case of using standard compound feeds in the feeding of laying quails [69,70,71,72,75,76,77,78,79,80,81,82,83,84].

### 4.3. Egg Quality Parameters

In the present research, the inclusion of whole lupine seeds in proportions of 20% and 25% in feed produced eggs with a lower weight than the control group that was fed with soybean meal. Similarly, a reduction in egg weight was reported when whole *L. albus* seeds were used in quantities between 180 and 300 g/kg [21] or *L. luteus* in quantities of 250 g/kg [68] in laying hen diets. Previously, Hammershøj and Steenfeldt [85] showed that hen egg weight was significantly reduced when whole seeds of *L. angustifolius* were used at a dose of 250 g/kg in feed. However, some research has shown that eggs with an insignificant weight difference can be obtained when blue lupine seeds are used up to 22% in feed [19,73] and even 30% [60].

Dehulled white lupine seeds used in the amount of 200 g/kg in the quail diets led to an increase in egg weight (*p* = 0.002) compared with the weight of eggs from quails with a diet with whole lupine seeds (200 g/kg), and this increase in egg weight was similar to the egg weight obtained in the control group. These findings are in agreement with the results of Laudadio and Tufarelli [37], who obtained eggs with a mean weight similar to the control group (without lupine) when using 18% dehulled white lupine seeds in the laying hen diets. Lee et al. [20], using dehulled blue lupine seeds for laying hen diets (150 g/kg), reported that the weight of the obtained eggs was not affected, compared with the control group, in which soybean meal was used.

Dehulling white lupine seeds did not significantly affect the proportion of egg albumen (*p* = 0.064) and yolk (*p* = 0.113) in the structure of the whole egg, but dehulling led to a reduction (*p* = 0.000) in the eggshell proportion and its thickness. Research by Laudadio and Tufarelli [37] has shown, on the other hand, that 18% dehulled white lupine used in the feeding of laying hens did not influence the proportion of yolk and eggshell in the structure of the whole egg or the shell thickness. Other research has shown that using whole yellow lupine seeds [60,68] and blue lupine [19,73] in proportions of 20–25% in the laying hen diets led to obtaining eggs with physical quality parameters (% albumen; % yolk; % Haugh unit) similar to those groups without lupine. The reduction in the eggshell thickness found in the present research in groups that received lupine in feed is in agreement with the research of Rutkowski et al. [68] and Drazbo et al. [19] on laying hens that were administered different proportions of lupine seeds in the diets. The decrease in eggshell thickness is probably caused by the higher soluble NSP content of lupine diets, which led to decreased digestion and absorption of minerals in laying quails. Nguyen et al. [67] observed that xylanase supplementation of a wheat-based diet (high soluble NSP) improved the eggshell thickness of hens because of increased digestion and absorption of minerals from feed.

The weight of quail eggs obtained in this research were in the value range of 11.2–14.6 g usually reported for this species [75,76,77,80,81,82]. According to scientific data, the proportion of morphological components in the structure of the whole egg is the following: for albumen, 54.1–60.8%; for yolk, 29.3–35.1%; and for eggshell, 9.0–16.1% [71,72,74,76,84]. Our results for these parameters are within the limits of the previously reported values.

### 4.4. Chemical Composition and Nutritional Quality of Eggs

Dehulling white lupine did not influence (*p* > 0.05) the raw chemical composition of quail eggs—namely, the albumen content in crude protein and the yolk in ether extract and crude protein. Similarly, Lee et al. [20] reported that by administration of dehulled blue lupine seeds in the laying hen diets there was no influence on the egg protein content.

The use of dehulled lupine seeds did not influence (*p* = 0.124) the egg cholesterol content, even if there was a tendency to decrease the cholesterol level in eggs from quails fed with whole or dehulled lupine diets. Our results are in agreement with those reported by Krawczyk et al. [60], who did not find any changes in the egg cholesterol content when whole seeds of *L. luteus* were used in the laying hen feed (300 g/kg), compared with the control group (based on soybean meal).

Dehulling white lupine seeds and their use in the quail diet led to a decrease in SFA concentration (35.80% vs. 38.06% of FAME) and an increase in the UFA level in the structure of yolk fats (63.80% vs. 61.39% of FAME). Additionally, the egg yolks from the groups of quails in which diets used the white lupine seeds (whole and dehulled) showed a higher level of MUFA and PUFA when compared with the control group (without lupine). These findings are also reported by Drazbo et al. [19], where blue lupine seeds were used in an amount of up to 200 g/kg in the diets of laying hens. The increase in MUFA and PUFA deposition in egg yolks, found when lupine seeds (whole and dehulled) were included in quail diets, may have been due to the rich content of lupine seeds in unsaturated fatty acids, especially in oleic (C18:1 *n*-9) and linoleic acids (C18:3 *n*-6). These unsaturated fatty acids were well represented quantitatively in the analyzed egg yolks of quails. These conclusions can be supported by research conducted recently by Timová et al. [86], who used white lupine seeds in the feed of laying hens to improve the quality of fats from egg yolks, obtaining a significant decrease in SFA and also an increase in PUFA content. Similar to our results, Krawczyk et al. [60] reported that the majority of fatty acids from egg yolks from laying hens fed with yellow lupine (up to 300 g/kg feed) were oleic (C18:1 *n*-9), palmitic (C16:0), and linoleic acids (C18:3 *n*-6). Moreover, the authors reported a decrease (*p* < 0.05) in the palmitic fatty acid level (C16:0) and an increase (*p* < 0.05) in the *n*-6 PUFA fatty acids level when lupine seeds were used in laying hen diets, similar to results also found in our research. Arachidonic fatty acid (C20: 4 *n*-6), which is a precursor to linoleic acid (C18:2 *n*-6), was found in higher proportions (*p* < 0.05) in egg yolks provided from lupine-fed quails (WLS_20_ and DLS_20_), compared with the quails fed diets without lupine (C). The presence of arachidonic fatty acid is considered important for consumer health because it contributes to increasing the proportion of unsaturated fatty acids to the detriment of saturated ones, which have a negative effect on the cardiovascular system in humans [87,88].

The nutritional quality of the fats from the egg yolks of quails was highlighted through the favorable sanogenic lipid indices, obtained when the whole lupine seeds and especially dehulled seeds were used in quail diets. The results highlight that these fats are characterized by a high level of polyunsaturated fatty acids, especially *n*-6 and *n*-3. The polyunsaturation index (PI) of yolk fats was higher when using dehulled lupine seeds (DLS_20_), compared with the situation without lupine (C) in quail feed (16.85 vs. 13.02) (*p* < 0.05). The use of lupine seeds (whole and dehulled) negatively influenced the *n*-6/*n*-3 FA ratio because it increased the concentration of *n*-6 FA in the structure of yolk fats because of linoleic acid, which was well represented in the fats of white lupine seeds. For a rational human nutrition, the ratio *n*-6/*n*-3 must be less than 4:1 for human diet [88], while in our study this ratio was much higher, between 6.63:1 in the control group and 10.63:1 to 16.77:1 in the DLS_20_ and WLS_20_ groups, respectively. Similar findings were reported by Krawczyk et al. [60] when using whole seeds of yellow lupine in the laying hen diets. The *n*-6/*n*-3 FA ratio obtained in our study was close to that reported by Krawczyk et al. [60] (16.77 vs. 12.67), ensuring a similar amount of whole lupine seeds in the laying hen feed (200 g/kg feed). Dehulling of lupine seeds had a positive influence on the *n*-6/*n*-3 FA ratio because the value of this quality index of yolk fats was reduced by 36.6% compared with the use of whole seeds (16.77 vs. 10.63). This is due to the decrease in the linolenic acid content in the fats structure of lupine after dehulling (*p* ˂ 0.001).

A decrease in the atherogenic (AI) and thrombogenic (TI) indices values was similarly recorded for laying hens when whole lupine seeds were included in the feed up to 200 g/kg [60,61]. The lower level of these indices positively influences human cardiovascular system health. The higher h/H (hypocholesterolemic/Hypercholesterolemic) index obtained for the groups in which white lupine seeds were used in quail feed was also observed by Criste et al. [61] when using white lupine in laying hen diets. The high value of the h/H index expresses the potential of fats to reduce the plasma cholesterol level due to increased intake of omega-3 fatty acids to the detriment of saturated fatty acids, which have a hypercholesterolemic effect [55,56].

### 4.5. Carotenoids Content in Egg Yolk

The use of whole and dehulled white lupine seeds in the quail diets led to eggs with a higher carotenoid content (*p* < 0.001) compared with eggs provided from the group without lupine. Higher concentrations of lutein, zeaxanthin, and canthaxanthin (*p* < 0.05) in egg yolks from WLS_20_ and DLS_20_ were due to the diet with lupine seeds.

Egg color is an important issue for consumers, and the color intensity is directly influenced by the natural pigments from the components of the feed compound [89,90]. These pigments are represented by carotenoids such as zeaxanthin, lutein, cantaxanthin, cryptoxanatin, or astaxanthine [20]. The increase in yolk color intensity (*p* < 0.001) in the eggs of quails that benefited from lupine in the diet (WLS_20_ and DLS_20_) was justified by the presence of carotenoids in lupine seeds, which led to the enrichment of egg yolk by carotenoids with antioxidant qualities [61,90,91]. On the other hand, some studies demonstrated the possibility of including natural ingredients rich in carotenoids (tomato powder rich in lycopene) in the diet of quails [92] and laying hens [89]. As a result, a significant increase in these carotenoids in yolk was achieved.

In the present research, the high carotenoid content of egg yolks was associated with a significantly higher color intensity in the lupine-fed quail groups. Similar to our results, Laudadio and Tufarelli [37] reported that dehulled white lupine seeds used in laying hen diets determined a significant increase in the yolk color intensity from 11.15 units (at diet without lupine) to 12.19 units (at diet with 180 g/kg dehulled lupine). Other research also proved an increase in yolk color intensity when whole seeds of *L. luteus* [60,69] and *L. angustifolius* [19,73,85] were included in feed (100–300 g/kg).

## 5. Conclusions

The inclusion of white lupine seeds (*L. albus*, cv. Amiga) at a rate of 20% whole seeds or 25% dehulled seeds in feed could provide an adequate source of protein for laying quails, without any negative effect on performance. The present study indicates the feasibility of supplementing diets with lupine for improving the quality of egg yolks by increasing the content of omega-3 FA and carotenoids, although such supplementation appears to have a negative effect on eggshell thickness. Further studies are needed to investigate the possibility of including higher amounts of white lupine (whole or dehulled seeds) in the diet of laying quails by using enzymes that degrade NSP.

## Figures and Tables

**Table 2:** Dehulling influence on the raw chemical composition of *L. albus* seeds (cv. Amiga), from low-alkaloid varieties (% of Dry Matter).

Specification (*n* = 5)	Whole Lupine Seeds	Dehulled Lupine Seeds	Tukey Test
	Mean ± sd	Mean ± sd	*p*-Value
Organic Matter	96.03 ± 0.03	96.10 ± 0.09	0.201
Crude ash	3.97 ± 0.03	3.90 ± 0.09	0.202
Crude protein	42.98 ± 0.49	51.83 ± 0.27	0.001
Ether extract	10.69 ± 0.31	11.90 ± 0.53	0.002
Crude fiber	14.01 ± 0.81	4.40 ± 0.19	0.001
N-FE extract	28.36 ± 1.03	27.97 ± 0.27	0.463
AME_N_ (kcal/kg DM)	3127.82 ± 71.87	4049.65 ± 40.76	0.001

sd, standard deviation. N-FE, nitrogen-free extract (calculated values: 100% − CP% − EE% − CF% − CA%); OM = 100 − CA%; AME_N_, nitrogen-corrected metabolizable energy, calculated according to Sibbald [46].

**Table 3:** Influence of dehulling *L. albus* (cultivar Amiga) seeds on the fatty acids content of fats (% of FAME).

Fatty Acids (*n* = 5)	Whole Lupine Seeds	Dehulled Lupine Seeds	Tukey Test
	Mean ± sd	Mean ± sd	*p*-Value
Miristic Acid (C14:0)	0.27 ± 0.02	0.21 ± 0.01	0.001
Pentadecanoic Acid (C15:0)	0.13 ± 0.01	0.09 ± 0.01	0.001
Palmitic Acid (C16:0)	10.46 ± 0.18	10.3 ± 0.14	0.148
Palmitoleic Acid (C16:1)	0.67 ± 0.04	0.65 ± 0.01	0.391
Stearic Acid (C18:0)	2.61 ± 0.09	2.58 ± 0.01	0.497
Oleic-*cis* Acid (C18:1 *n*-9)	50.72 ± 0.09	51.04 ± 0.1	0.001
Linoleic Acid (C18:2 *n*-6)	14.51 ± 0.09	13.79 ± 0.18	0.001
γ-linolenic Acid (C18:3 *n*-6)	0.07 ± 0.09	0.09 ± 0.05	0.811
α-linolenic Acid (C18:3 *n*-3)	13.09 ± 0.17	13.26 ± 0.17	0.161
Arachidic Acid (C20:0)	0.95 ± 0.07	0.97 ± 0.02	0.541
Eicosenoic Acid (C20:1 *n*-9)	3.01 ± 0.03	3.39 ± 0.25	0.008
Eicosadienoic Acid (C20:2 *n*-6)	0.19 ± 0.01	0.18 ± 0.01	0.056
Behenic Acid (C22:0)	2.07 ± 0.02	2.21 ± 0.05	0.001
Erucic Acid (C22:1 *n*-9)	0.73 ± 0.02	0.7 ± 0.05	0.289
Eicosapentaenoic Acid (C20:5 *n*-3)	0.28 ± 0.02	0.18 ± 0.07	0.015
Other fatty acids	0.28 ± 0.05	0.38 ± 0.38	0.004
Σ SFA	16.46 ± 0.28	16.36 ± 0.09	0.466
Σ MUFA	55.12 ± 0.78	55.78 ± 0.14	0.001
Σ PUFA	28.14 ± 0.17	27.48 ± 0.19	0.001
Σ UFA	83.26 ± 0.29	83.28 ± 0.10	0.881

sd, standard deviation. FAME, fatty acids methyl esters; SFA, saturated fatty acids; MUFA, monounsaturated fatty acids; PUFA, polyunsaturated fatty acids; UFA, unsaturated fatty acids.

**Table 4:** Influence of dehulling *L. albus* (cv. Amiga) seeds on the amino acids content (g/16 g N).

Amino Acids (*n* = 6)	Whole Lupine Seeds	Dehulled Lupine Seeds	Tukey Test
	Mean ± sd	Mean ± sd	*p*-Value
Essential amino acids			
Lysine	4.99 ± 0.02	5.73 ± 0.14	0.001
Methionine	0.48 ± 0.01	0.55 ± 0.01	0.001
Tryptophan	0.47 ± 0.01	0.51 ± 0.01	0.001
Histidine	4.84 ± 0.18	3.61 ± 0.05	0.001
Valine	3.24 ± 0.02	3.38 ± 0.02	0.035
Phenylalanine	2.14 ± 0.03	1.06 ± 0.02	0.001
Isoleucine	2.33 ± 0.06	1.64 ± 0.03	0.001
Leucine	5.76 ± 0.04	4.01 ± 0.06	0.001
Arginine	9.42 ± 0.04	10.06 ± 0.03	0.001
Non-essential amino acids			
Serine + aspartic acid	7.55 ± 0.10	9.03 ± 0.11	0.001
Glutamine	31.66 ± 0.55	36.64 ± 0.25	0.001
Proline	6.29 ± 0.09	7.99 ± 0.06	0.001
Alanine	2.08 ± 0.04	5.54 ± 0.02	0.001

sd—standard deviation.

**Table 5:** Influence of using dehulled white lupine seeds in the laying quail diets on the performance responses.

Specification	C	Experimental Treatments (Mean ± sd)				ANOVA Two-Way	
		WLS_20_	WLS_25_	DLS_20_	DLS_25_	F-Value	*p*-Value
Initial weight of quails (g/quail)	288.26 ± 17.90 ^a^	287.47 ± 19.67 ^a^	290.77 ± 15.03 ^a^	287.22 ± 17.79 ^a^	286.86 ± 14.52 ^a^	0.312	0.869
Final weight of quails (g/quail)	313.76 ± 26.73 ^a^	297.03 ± 24.72 ^b^	297.87 ± 23.52 ^b^	301.16 ± 24.45 ^ab^	297.77 ± 27.81 ^b^	2.981	0.021
Daily feed intake (g of feed/quail)	32.09 ± 1.12 ^a^	32.54 ± 1.33 ^a^	32.62 ± 1.21 ^a^	32.43 ± 1.18 ^a^	32.47 ± 1.43 ^a^	0.795	0.530
Laying rate (%)	92.41 ± 8.62 ^a^	91.65 ± 9.76 ^ab^	89.78 ± 9.69 ^b^	92.59 ± 9.03 ^a^	91.29 ± 10.07 ^ab^	4.132	0.002
Feed conversion ratio: kg feed/kg egg mass	2.68 ± 0.14 ^a^	2.78 ± 0.20 ^ab^	2.82 ± 0.16 ^b^	2.70 ± 0.16 ^a^	2.77 ± 0.21 ^ab^	5.983	0.000
kg feed/dozen eggs	0.417 ± 0.02 ^a^	0.426 ± 0.02 ^ab^	0.436 ± 0.03 ^b^	0.420 ± 0.03 ^ab^	0.427 ± 0.02 ^ab^	1.962	0.018

sd, standard deviation; ^a–c^, different superscripts in the same row show significant differences (*p* < 0.05). C, control group, without lupine; WLS_20_, experimental diet with 200 g/kg whole lupine seeds; WLS_25_, experimental diet with 250 g/kg whole lupine seeds; DLS_20_, experimental diet with 200 g/kg dehulled lupine seeds; DLS_25_, experimental diet with 250 g/kg dehulled lupine seeds.

**Table 6:** Influence of using dehulled white lupine seeds in the laying quail diets on the physical parameters of the egg.

Specification	C	Experimental Treatments (Mean ± sd)				ANOVA Two-Way	
		WLS_20_	WLS_25_	DLS_20_	DLS_25_	F-Value	*p*-Value
Egg weight (g)	13.02 ± 0.07 ^a^	12.89 ± 0.14 ^b^	12.87 ± 0.13 ^b^	13.04 ± 0.20 ^a^	12.90 ± 0.15 ^ab^	1.759	0.002
Albumen (%)	58.00 ± 1.80 ^a^	58.33 ± 1.94 ^a^	58.35 ± 1.66 ^a^	58.26 ± 1.85 ^a^	57.76 ± 1.65 ^a^	3.573	0.064
Yolk (%)	29.81 ± 1.44 ^a^	29.88 ± 1.66 ^a^	29.60 ± 1.42 ^a^	29.66 ± 1.80 ^a^	30.08 ± 1.44 ^a^	1.877	0.113
Shell (%)	12.18 ± 0.85 ^a^	11.81 ± 0.76 ^b^	12.05 ± 0.85 ^b^	12.10 ± 0.93 ^ab^	12.16 ± 0.89 ^a^	7.725	0.000
Shell thickness (mm)	0.212 ± 0.01 ^a^	0.197 ± 0.01 ^c^	0.194 ± 0.01 ^c^	0.203 ± 0.01 ^b^	0.198 ± 0.01 ^bc^	4.081	0.000

sd, standard deviation; ^a–c^, different superscript letters in the same row are significant differences (*p* < 0.05). C, control group, without lupine; WLS_20_, experimental diet with 200 g/kg whole lupine seeds; WLS_25_, experimental diet with 250 g/kg whole lupine seeds; DLS_20_, experimental diet with 200 g/kg dehulled lupine seeds; DLS_25_, experimental diet with 250 g/kg dehulled lupine seeds.

**Table 7:** Influence of dehulling white lupine seeds used in the laying quail diets on the fatty acids profile of fats from egg yolk (% of FAME).

Fatty Acids	C	Experimental Treatments (Mean ± sd)		ANOVA Single-Way	
		WLS_20_	DLS_20_	F-Value	*p*-Value
Miristic Acid (C14:0)	0.30 ± 0.03 ^a^	0.30 ± 0.01 ^a^	0.29 ± 0.02 ^a^	0.40	0.680
Miristoleic Acid (C14:1)	0.04 ± 0.01 ^a^	0.03 ± 0.01 ^a^	0.04 ± 0.01 ^a^	2.90	0.094
Pentadecanoic Acid (C15:0)	0.03 ± 0.01 ^a^	0.03 ± 0.01 ^a^	0.04 ± 0.01 ^a^	17.49	0.089
Pentadecenoic Acid (C15:1)	0.13 ± 0.03 ^a^	0.20 ± 0.13 ^a^	0.14 ± 0.06 ^a^	1.34	0.298
Palmitic Acid (C16:0)	25.84 ± 0.41 ^a^	23.42 ± 2.41 ^b^	21.38 ± 0.12 ^b^	4.33	0.038
Palmitoleic Acid (C16:1)	2.92 ± 0.25 ^a^	2.90 ± 0.33 ^a^	3.50 ± 0.08 ^b^	9.84	0.003
Heptadecanoic Acid (C17:0)	0.11 ± 0.02 ^a^	0.10 ± 0.01 ^a^	0.15 ± 0.01 ^b^	26.82	0.000
Heptadecenoic Acid (C17:1)	0.18 ± 0.050 ^a^	0.21 ± 0.03 ^a^	0.18 ± 0.07 ^a^	0.78	0.482
Stearic Acid (C18:0)	15.91 ± 0.50 ^a^	14.22 ± 0.99 ^a^	13.95 ± 0.12 ^a^	0.35	0.714
Oleic Acid (C18:1 *n*-9)	32.26 ± 1.12 ^a^	33.92 ± 1.04 ^ab^	34.66 ± 0.22 ^b^	30.61	0.000
Linoleic Acid (C18:2 *n*-6)	12.49 ± 1.55 ^a^	15.31 ± 1.63 ^b^	16.31 ± 0.22 ^b^	25.15	0.000
Γ-linolenic Acid (C18:3 *n*-6)	0.18 ± 0.03 ^a^	0.27 ± 0.01 ^b^	0.27 ± 0.02 ^b^	31.84	0.000
α-linolenic Acid (C18:3 *n*-3)	0.27 ± 0.03 ^a^	0.12 ± 0.03 ^b^	0.27 ± 0.01 ^a^	55.79	0.000
Eicosadienoic Acid (C20:2 *n*-6)	0.17 ± 0.05 ^a^	0.13 ± 0.03 ^a^	0.17 ± 0.03 ^a^	2.61	0.115
Eicosatrienoic Acid (C20:3 *n*-6)	0.23 ± 0.01 ^a^	0.20 ± 0.05 ^ab^	0.15 ± 0.02 ^b^	9.27	0.004
Eicosatrienoic Acid (C20:3 *n*-3)	0.22 ± 0.02 ^a^	0.23 ± 0.01 ^a^	0.20 ± 0.04 ^a^	1.68	0.227
Erucic Acid (C22:1 *n*-9)	0.04 ± 0.01 ^a^	0.04 ± 0.01 ^a^	0.05 ± 0.01 ^a^	3.52	0.063
Arachidonic Acid (C20:4 *n*-6)	5.02 ± 0.12 ^a^	5.36 ± 0.49 ^b^	5.16 ± 0.04 ^b^	14.75	0.001
Nervonic Acid (C24:1 *n*-9)	0.32 ± 0.02 ^a^	0.25 ± 0.03 ^b^	0.16 ± 0.03 ^c^	39.02	0.000
Docosatetraenoic Acid (C22:4 *n*-6)	0.55 ± 0.15 ^a^	1.41 ± 0.17 ^b^	0.84 ± 0.04 ^a^	53.27	0.000
Docosapentaenoic Acid (C22:5 *n*-3)	0.27 ± 0.02 ^a^	0.14 ± 0.04 ^b^	0.25 ± 0.04 ^a^	18.89	0.000
Docosahexaenoic Acid (C22:6 *n*-3)	2.06 ± 0.05 ^a^	0.86 ± 0.18 ^b^	1.44 ± 0.07 ^c^	131.26	0.000
Other fatty acids	0.47 ± 0.28 ^a^	0.55 ± 0.18 ^a^	0.40 ± 0.17 ^a^	0.10	0.908
Σ SFA	42.19 ± 0.74 ^a^	38.06 ± 2.02 ^b^	35.80 ± 2.02 ^c^	5.16	0.024
Σ UFA	57.35 ± 0.68 ^s^	61.39 ± 1.97 ^b^	63.80 ± 1.97 ^c^	5.77	0.018
Σ MUFA	35.88 ± 1.33 ^a^	37.56 ± 1.35 ^ab^	38.74 ± 1.35 ^b^	24.33	0.000
Σ PUFA	21.47 ± 1.78 ^a^	23.83 ± 2.45 ^b^	25.06 ± 2.45 ^b^	14.06	0.001
*n*-3 FA	2.82 ± 0.08 ^a^	1.34 ± 0.22 ^b^	2.16 ± 0.22 ^c^	105.57	0.000
*n*-6 FA	18.65 ± 1.75 ^a^	22.48 ± 2.26 ^b^	22.89 ± 2.26 ^b^	23.17	0.000
HFA	26.14 ± 0.38 ^a^	23.71 ± 2.42 ^b^	21.66 ± 2.42 ^b^	4.37	0.038
hFA	53.72 ± 0.74 ^a^	52.74 ± 2.02 ^a^	59.72 ± 2.02 ^b^	7.26	0.009

sd, standard deviation; ^a–c^, different superscripts in the same row show significant differences (*p* < 0.05). C, control group, without lupine; WLS_20_, experimental diet with 200 g/kg whole lupine seeds; DLS_20_, experimental diet with 200 g/kg dehulled lupine seeds. FA, fatty acids; FAME, fatty acids methyl esters; SFA, saturated fatty acids; MUFA, monounsaturated fatty acids; PUFA, polyunsaturated fatty acids; UFA, unsaturated fatty acids; *n*-3, omega 3 fatty acids; *n*-6, omega 6 fatty acids; HFA, hypercholesterolemic fatty acids (C14:0 + C16:0); hFA, hypocholesterolemic fatty acids (C18:1 + polyunsaturated FA).

**Table 8:** Influence of dehulling white lupine seeds used in the laying quail diets on the lipid quality indices of egg fats.

Indices	C	Experimental Treatments (Mean ± sd)		ANOVA Single Way	
		WLS_20_	DLS_20_	F-value	*p*-Value
PUFA/SFA	0.51 ± 0.06 ^a^	0.63 ± 0.12 ^ab^	0.70 ± 0.12 ^b^	8.10	0.006
MUFA/SFA	0.85 ± 0.02 ^a^	0.99 ± 0.07 ^b^	1.08 ± 0.07 ^c^	13.29	0.001
UFA/SFA	1.36 ± 0.05 ^a^	1.61 ± 0.17 ^b^	1.79 ± 0.17 ^c^	4.88	0.028
*n*-6/*n*-3 FA	6.63 ± 1.32 ^a^	16.77 ± 0.67 ^b^	10.63 ± 0.67 ^c^	222.52	0.000
h/H	2.06 ± 0.07 ^a^	2.44 ± 0.59 ^ab^	2.80 ± 0.59 ^b^	3.68	0.057
PI	13.02 ± 1.60 ^a^	15.26 ± 1.69 ^ab^	16.85 ± 1.69 ^b^	22.60	0.000
AI	0.47 ± 0.02 ^a^	0.40 ± 0.05 ^b^	0.35 ± 0.05 ^b^	21.59	0.000
TI	1.17 ± 0.03 ^a^	1.11 ± 0.08 ^a^	0.95 ± 0.08 ^b^	20.87	0.000
HPI	2.12 ± 0.05 ^a^	2.50 ± 0.55 ^ab^	2.87 ± 0.55 ^b^	10.68	0.002

sd, standard deviation; ^a–c^, different superscripts in the same row show significant differences (*p* < 0.05). C, control group, without lupine; WLS_20_, experimental diet with 200 g/kg whole lupine seeds; DLS_20_, experimental diet with 200 g/kg dehulled lupine seeds. SFA, saturated fatty acids; MUFA, monounsaturated fatty acids; PUFA, polyunsaturated fatty acids; UFA, unsaturated fatty acids; *n*-3, omega 3 fatty acids; *n*-6, omega 6 fatty acids; PI, polyunsaturated index; AI, atherogenic index; TI, thrombogenic index; h/H, hypocholesterolemic/Hypercholesterolemic FA; HPI (health promotion index) = (*n*-3 PUFA + *n*-6 PUFA + MUFA) ÷ [C12:0 + (4 × C14:0) + C16:0].

**Table 9:** Influence of dehulling white lupine seeds on the carotenoids content of egg yolk (µg/g fresh weight).

Yolk Carotenoids	C	Experimental Treatments (Mean ± sd)		ANOVA Single-Way	
		WLS_20_	DLS_20_	F-Value	*p*-Value
Lutein	4.28 ± 1.36 ^a^	7.01 ± 0.50 ^b^	6.01 ± 0.76 ^b^	10.746	0.002
Zeaxanthin	6.02 ± 1.52 ^a^	9.31 ± 1.04 ^b^	8.38 ± 0.18 ^b^	12.623	0.001
Canthaxanthin	3.08 ± 0.66 ^a^	5.55 ± 2.13 ^b^	3.73 ± 0.84 ^ab^	4.325	0.039
ß-cryptoxanine	0.53 ± 0.11 ^a^	0.93 ± 0.35 ^b^	0.57 ± 0.07 ^ab^	5.227	0.023
Total carotenoids	13.91 ± 3.14 ^a^	22.8 ± 2.13 ^b^	18.63 ± 1.20 ^b^	18.804	0.000
Yolk color intensity	8.86 ± 1.14 ^a^	12.77 ± 3.14 ^b^	12.14 ± 3.14 ^b^	120.00	0.000

sd, standard deviation; ^a,b^: different superscripts in the same row show significant differences (*p* < 0.05). C, control group, without lupine; WLS_20_, experimental diet with 200 g/kg whole lupine seeds; DLS_20_, experimental diet with 200 g/kg dehulled lupine seeds.

## Data Availability

The data supporting the reported results are in the possession of the authors.
